# Infection

**Published:** 1892-09

**Authors:** H. L. Ambler

**Affiliations:** Cleveland, O.


### THE DENTAL REGISTER.

VOL. XLVI.] SEPTEMBER, 1892. [No. 9.

### COMMUNICATIONS.

### Infection.

BY H. L. AMBLER, D.D.S., M.D., CLEVELAND, O.

Read before the Northern Ohio Dental Society, Cleveland, May, 1892.

The paper which I present for your consideration consists of a few thoughts on Infection by leading thinkers.

The science of bacteriology has so grown and developed within the past few years as to reach with almost equal importance the domains of medicine and surgery, and to touch most of the therapeutical questions involved in the treatment of medical and surgical affections. It has an important bearing on the prevention and treatment of disease, while it has a connection, also, with the period of convalescence. Bacteria as a cause of disease, is taken for granted, and in the mouth their growth is rapid, infectious varieties have been found here and they are easily propagated; temperature, air, water and food, give them the very best opportunity for flourishing. There are over one hundred different species present at one time or another in the human mouth, but some of them do no harm. In two days a bacterium will multiply to the almost incredible number of 281,-500,000,000, and it will require 636,000,000 to weigh one gramme. Two hundred years ago Leeuwenhoek described and illustrated a germ, spiral in form, which he found in the mouth, the entire group was classed under the term microbe, and he said they could not penetrate the dentine, except so far as it had been softened by some means.

Pathogenic organisms are found in putrescible matter, and also have power to attack living tissues; these forms, including the pyogenic bacteria, are of great importance to dentists. Non-pathogenic organisms can only live and grow in dead and dying matter. When the system is in a debilitated condition these germs plant themselves and grow most rapidly. At times it is not absolutely necessary to have an abraded mucous surface in order to permit of infection. The changes of putrefaction are dependent upon a living organism and a nidus for its life; substances interfering with the organisms causing these changes are termed antiseptics. Suppuration is one of the phenomena attending the efforts of certain germs to invade the tissues; prevent invasion, and you prevent suppuration, and disinfection is any means put into use for the destruction of these germs. Antiseptic surgery was started on the foundation established by Pasteur, that germ-laden air is directly instrumental in causing putrefaction and the fundamental principle of modern surgery is antisepsis. Fracture of the jaw with penetration of the soft tissues, extraction of teeth, gum-lancing, dead pulps, ulcers, abscesses, broken teeth and poorly fitting plates present opportunities for infection, but we do not always find infection following such injuries; if the bacteria are scarce, the repair cells are victorious and no disease ensues. The chances of infection from any cause whatever should always be thought of and precautions used. The ear, nose, throat, parotid gland and antrum, may become seats of infection which will more or less affect the mouth, or infection of the antrum may take place from the nose, or from inflammation of the carious or abscessed teeth. I impress it on patients to keep the mouth in an aseptic condition by the use of the brush and antiseptic washes, such as diluted listerine or peroxide of hydrogen which are germicides. In manipulations be as cleanly as possible, this includes thoroughly cleansing the hands and instruments with antiseptics. It has been demonstrated that infectious germs are present on the hands and under the nails, but thorough washing with soap and brush will remove all easily detachable epithelial germs, after which rinse in the following: one dram bichloride of mercury, one ounce of alcohol mixed; one-half of a teaspoonful of this mixture to one pint of water makes about one of mercury to 2,000 water; this can be further diluted if it does not agree with the hands, as some claim that 1 to 5,000 is of sufficient strength. Grosvenor and Richards manufacture tablets composed of equal parts of bichloride of mercury and chloride of ammonium; one of these dissolved in a pint of water makes a 1 to 1,000 solution, but the strength can be altered at will; clean the nails before using either preparation, and afterwards rinse the hands in pure water. In abdominal operations the hands are immersed in a saturated solution of permanganate of potash until they are mahogany red, then transferred to a saturated solution of oxalic acid until they become pink, then washed in warm sterilized water, this renders them completely aseptic. Instruments can be sterilized in boiling water containing five per cent. of carbolic acid, or in the bichloride mixture; but if the latter is used great care must be taken or they will become rusty; baking them for a few moments at a temperature of 300° in a gas stove is highly recommended; moist heat applied for twenty minutes accomplishes sterilization, and it can be successfully applied by Arnold's steam sterilizer. Instruments are not apt to lose polish or rust unless cooled before wiping dry. Organic matter yields last to the destructive influence of micro-organisms, and where softened dentine is left over a pulp and the cavity filled we may cover in microbes and they may produce products which will destroy the pulp, thus producing infection. In such cases do not use any medicament which will produce coagulation, but desiccate the softened dentine with warm air and apply oil of cassia which is diffusible, penetrating and will destroy the microbes; some prefer oil of cassia and oil of cloves, equal parts; myrtol is also recommended because it is non-irritating and a powerful antiseptic; lysol, which is soluble in water, is used for the same purpose. A decoction of cinnamon ought to be taken freely by persons living in places affected by typhoid or cholera. In the oldest medical prescriptions for infectious diseases, cinnamon was a prominent ingredient, and it was in great request during the plague of London; and it is claimed by many that oil of cassia will destroy microbes as effectually and rapidly as bichloride of mercury. Pyæmia is infectious, for the products poured out by the micrococci, which are from $\frac{1}{10000}$ to $\frac{1}{25000}$ of an inch in diameter, poison the blood and the absorption and dissemination of the poison give rise to general disease and also abscess. While in his office Dr. E. accidentally inoculated himself and in a short time had an abscess large as his hand. Dr. B. unfortunately cut his finger with an instrument he had been using in a decayed tooth, the result was pyæmia, which incapacitated him for several weeks. We will surely be more successful in the treatment of lesions of the mouth and teeth by careful attention to antisepsis. Where the saliva is in a septic condition, caused by pyogenic bacteria from alveolar abscess, it can be rendered inert by using a solution of one part of peroxide of hydrogen and two parts of water; a spoonful held in the mouth for five minutes will destroy a large number of germs and oxidize minute particles of dead organic matter which have not been removed by floss and brush. Infection of the general system may be produced by a diseased condition of the mouth, as the microbes which produce pneumonia, erysipela, and septicæmia, have been found in the oral cavity. Great numbers of germs are constantly being swallowed with the saliva, and some of them are ready to do us harm. The cleaner the mouth is kept the more slowly will microbes develop. Tooth-powders and tooth-washes should be strongly impregnated with oil of cassia which will act as a prophylactic.

